# Evaluating the impact of patient-reported outcome measures on depression and anxiety levels in people with multiple sclerosis: a study protocol for a randomized controlled trial

**DOI:** 10.1186/s12883-023-03090-0

**Published:** 2023-02-02

**Authors:** Nathan Y. Chu, Kaitlyn E. Watson, Yazid N. Al Hamarneh, Lily Yushko, Ross T. Tsuyuki, Penelope Smyth

**Affiliations:** 1 Department of Medicine, Division of Neurology, University of Alberta, 7-132B Clinical Sciences Building, 8440 112 Street NW, Edmonton, AB T6G 2B7 Canada; 2 EPICORE (Epidemiology Coordinating and Research) Centre, Department of Medicine, University of Alberta, Edmonton, AB Canada; 3 Department of Pharmacology, University of Alberta, Edmonton, AB Canada; 4 Department of Medicine, University of Alberta, Edmonton, AB Canada

**Keywords:** Multiple sclerosis, Patient-reported outcome measures, Patient satisfaction, Depression, Anxiety, HADS, Healthcare delivery

## Abstract

**Background:**

Multiple sclerosis (MS) is a chronic disease affecting multiple functional aspects of patients’ lives. Depression and anxiety are common amongst persons with MS (PwMS). There has been an interest in utilizing patient-reported outcome measures (PROMs) to capture and systematically assess patient’s perceptions of their MS experience in addition to other clinical measures, but PROMs are not usually collected in routine clinical practice. Therefore, this study aims to systematically incorporate periodic electronically administered PROMs into the care of PwMS to evaluate its effects on depression and anxiety.

**Methods:**

A randomized controlled trial will be conducted with patients allocated 1:1 to either intervention or conservative treatment groups. Patients in the intervention group will complete PROMs at the start of the study and then every 6 months for 1 year, in addition to having their MS healthcare provider prompted to view their scores. The conservative treatment group will complete PROMs at the start of the study and again after 12 months, and their neurologist will not be able to view their scores. For both groups, pre-determined critical PROM scores will trigger an alert to the patient’s MS provider. The difference in change in Hospital Anxiety and Depression Scale score between the intervention and conservative treatment groups at 12 months will be the primary outcome, along with difference in Consultation Satisfaction Questionnaire and CollaboRATE scores at 12 months, and proportion and type of healthcare provider intervention/alerts initiated by different PROMs as secondary outcomes.

**Discussion:**

This study will determine the feasibility of utilizing PROMs on an interval basis and its effects on the psychological well-being of PwMS. Findings of this study will provide evidence on use of PROMs in future MS clinical practice.

**Trial registration:**

This trial is registered at the National Institutes of Health United States National Library of Medicine, ClinicalTrials.gov NCT04979546. Registered on July 28, 2021.

**Supplementary Information:**

The online version contains supplementary material available at 10.1186/s12883-023-03090-0.

## Background

Multiple sclerosis (MS) is a disease that can affect physical, cognitive, psychological, and social functioning [1–3]. While physical symptoms and disability are typically the focus of routine care, there is an increasing recognition of psychiatric comorbidity in multiple sclerosis, particularly depression and anxiety; the presence of depression and anxiety are known to reduce quality of life in persons with MS (PwMS) [4–6]. A large meta-analysis including over 87,000 PwMS estimates prevalence of depression and anxiety at 30.5% and 22.1%, respectively [7], emphasizing the heightened burden of mental illness in this population. The downstream effects of depression and anxiety in MS are multifaceted beyond the negative impact upon quality of life, having been linked to nonadherence to disease modifying treatments [8], increased rate of hospitalizations [9], and increased mortality [10].

Patient-reported outcome measures (PROMs) give patients the opportunity to describe their symptoms and the impact of care in a consistent and systematic fashion [11], and the incorporation of PROMs into several large registries in North America and Europe highlights their increasing relevance in MS research [12–15]. PROMs are thought to be important in optimizing the management of PwMS in providing the patient’s perspective of their disease [16]. Additionally, use of PROMs between clinic visits could provide real-time information for healthcare providers, which is vital in a disease with episodic disability and fluctuating symptoms over time. Even though such information could help clinicians provide patient-centred care and patients track their progress, it is not usually collected on a routine basis due to time limitations and system issues within clinics, amongst other factors [17].

Electronic data capture could help overcome these difficulties, as patients will have the flexibility to provide the information from anywhere and the data will automatically be saved in an electronic database that can be accessed anytime by patients and their healthcare providers. This will allow patients to be fully informed about their condition, be more engaged in their care, track their progress, and receive any notifications or alerts regarding their care. It will also allow healthcare providers to systematically track the patient’s progress, better prepare for clinic visits and to better provide care based on patient’s individual needs. This patient-centred approach could enhance the care that PwMS receive. Regular use of PROMs also has potential for PwMS to provide information about their symptoms and quality of life (QoL) before their visits. This could free up time during clinic visits to address identified concerns and needs, and then come up with a management and support plan based on those needs.

In addition to offering data to providers, routine PROMs may potentially reinforce patient activation and engagement, which are related terms to describe a patient having adequate knowledge and ability to actively participate and manage their healthcare, and the interventions and behaviours demonstrating the former, respectively [18]. Patient activation is associated with improved health outcomes, enhanced healthcare experiences, and lower costs across different chronic diseases [18]. PROMs support patient activation and engagement by providing a platform for patients to voice the impact of their disease through the lens of their unique experiences and lifestyle [19]. Thus, there is considerable motivation to incorporate PROMs into routine clinical care, but among issues including logistical and technological challenges, the optimal timing and frequency of reporting PROMs is unknown [19–22].

Furthermore, using PROMs longitudinally is not known to affect mental health outcomes in PwMS by providing healthcare providers objective measures to track scores and intervene when needed. The present study seeks to fill this knowledge gap by examining the systematic incorporation of electronically administered PROMs into the care of PwMS and exploring their effects on depression and anxiety.

## Objectives

The aim of this study is to evaluate the systematic use of PROMS in PwMS in both an academic MS clinic and in community neurology office practices. Specifically, the goal is to examine the impact of regular utilization of PROMs on patients’ mental health outcomes and satisfaction with care, and the difference in those measures depending on PROM administration frequency. We hypothesize that:

1. Patients completing PROMs more frequently will have a greater improvement in depression and anxiety scores in PwMS.
2. Providers will initiate an intervention for a higher proportion of PwMS completing PROMs more frequently compared to usual care.
3. PwMS completing more frequent PROMs will have a greater satisfaction in care provided by their clinicians.

## Methods

This protocol is in accordance with Standard Protocol Items: Recommendations for Interventional Trials (SPIRIT) 2013 guidelines. Ethics approval was obtained by the University of Alberta Research Ethics Office (Health Research Ethics Board – Health Panel ID: Pro00111593).

### Study population

Participants will be PwMS managed by a neurologist or MS nurse practitioner in northern Alberta, Canada. This includes a tertiary care academic centre (the Kaye Edmonton Clinic – Northern Alberta MS Clinic affiliated with the University of Alberta in Edmonton, Alberta, Canada), a community hospital-based outpatient MS clinic (MS Clinic, Red Deer Regional Hospital in Red Deer, Alberta, Canada) and additional private community neurologist practices (Westmount Neurology Clinic, and Edmonton Specialists Clinic, both in Edmonton, Alberta, Canada).

Inclusion criteria are:

1. A confirmed diagnosis of MS by a qualified healthcare provider. All subtypes falling under the diagnosis of multiple sclerosis including relapsing–remitting, secondary progressive, and primary progressive, among others, are eligible to participate.
2. Active patient of an Alberta-based neurologist/MS nurse practitioner.
3. Able/willing to complete informed consent and electronic PROM questionnaires.
4. Able to use a computer/smartphone.
5. English-speaking.

Exclusion criteria are:

1. A suspected but not confirmed diagnosis of MS, a diagnosis of clinically/radiologically isolated syndrome, or a central nervous system inflammatory disorder other than MS.
2. PwMS not being managed by a participating neurologist/MS nurse practitioner.
3. Unwilling/unable to provide consent.
4. Unwilling/unable complete the electronic PROM questionnaires.
5. Cannot speak English.
6. Under the age of 18.

### Participant recruitment

Patients will be contacted and informed about the study via four primary methods:

1. Participating outpatient community neurologists/MS nurse practitioners will provide rosters of PwMS, and patients will be contacted via secured and encrypted email messaging.
2. All patients (*n* = 246) who previously participated in a related study by our group [23] will be contacted via email/phone number; as part of the aforementioned study, they have already consented to be contacted to participate in future research projects.
3. Patients with MS providers at the Northern Alberta MS Clinic will be contacted via secured messaging through MyChart® (Epic Systems Corporation©).
4. Advertisement posters (Supplementary Fig. 1) will be displayed in participating clinics. Patients will be able to access a link via Uniform Resource Locator (URL) or Quick Response (QR) code to provide informed consent and self-registration.

Health care providers (MS neurologists and nurse practitioners) will be identified by the researchers and contacted directly via email, telephone, or in-person conversation. Groups of providers (those based out of the Kaye Edmonton Clinic at the University of Alberta and community neurologists with private practices) will be given formal presentations on the research protocol and aims, and the extent of the providers’ participation.

### Patient enrolment

Interested patients responding to our recruitment methods will be given a link to the Research Electronic Data Capture (REDCap) tools and services at the University of Alberta, where they will be provided with further study information and asked to provide digital informed consent for self-registration (Supplementary Fig. 2). During self-registration, they will select their MS provider from a drop-down list. Our research team will then assign them to a Data Access Group (DAG) to enlist the patient in a roster based on their provider; this will allow each provider to confidentially view their own patients’ PROM scores throughout the study.

### Design

**Fig. 1:**
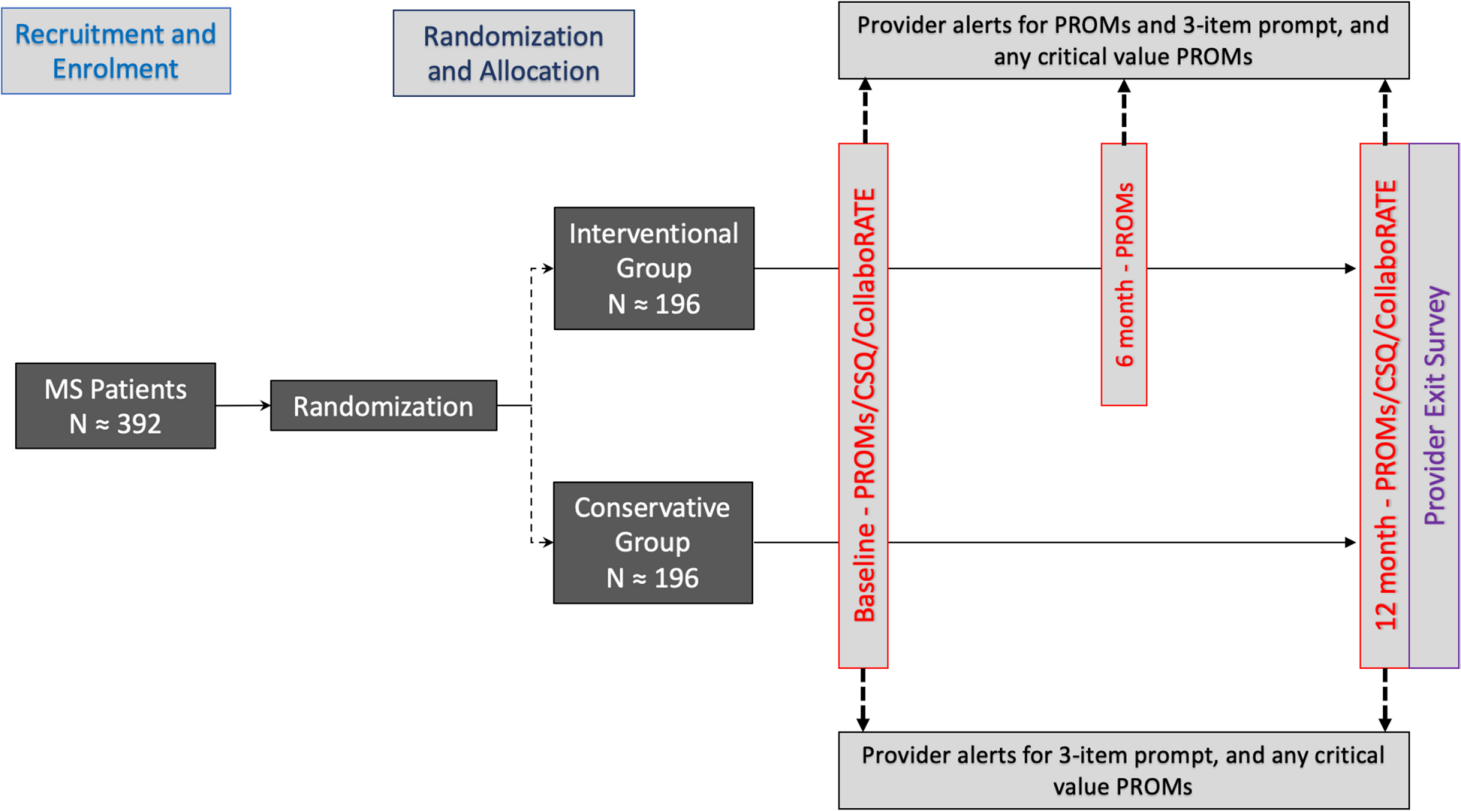
Study protocol timeline and flow chart. MS, multiple sclerosis; PROMs, patient reported outcome measures; CSQ; consultant satisfaction questionnaire

This study is a randomized controlled trial with the patient as the unit of randomization. Patients will be randomized electronically in a 1:1 ratio to the intervention or conservative groups at study enrolment using a native REDCap randomization module to ensure allocation concealment. The timeline of the study protocol is depicted in Fig. 1.

Participants are randomly assigned in a 1:1 ratio to one of two groups:

1. *Intervention group*: All participants randomized to the intervention group will be asked to complete the following validated questionnaires at baseline and approximately every 6 months for a total of 12 months:
  Hospital Anxiety and Depression Scale (HADS) score [24]
  Quality of life as measured by EuroQol five-dimensional questionnaire (EQ5D) [25]
  Fatigue as measured by Modified Fatigue Impact Scale (MFIS) [26]
  The Patient Determined Disease Steps (PDDS) [27]
  Patient Health Questionnaire-9 (PHQ-9) [28]
  Open ended text response to (limit 280 characters) “What are the top 3 things you would like your MS healthcare provider to know about you right now?” – to be referred as the 3-item prompt (3IP).
   Completed questionnaires will be sent to the treating provider via a secured link using encrypted email to review the corresponding scores and the text response to the 3IP.
2. *Conservative group*: All participants randomized to the conservative group will be asked to complete the same questionnaires as above, however only at baseline and at the end of the study at 12 months. Additionally, the treating provider will only be prompted to review the text response to the 3IP and will not be able to access the PROM questionnaire scores.

For both groups, providers will be able to document via REDCap whether they have reviewed the aforementioned items and their response/intervention, if applicable (Supplementary Fig. 3). Examples of provider responses would be phone calls to patients, scheduling earlier appointments, alterations to medication regimens, referral to another health or medical provider, and/or messaging the patient via secure email messaging system; a free text response will also be included to allow documentation of alternative interventions if required. Due to initial lack of provider engagement, providers will be incentivized with token of appreciation ($10 Amazon gift card) for each instance of documenting they have reviewed (and if applicable, enacted healthcare provider intervention in response to) their patients’ PROM scores and/or text responses to 3IP. There will be no difference in incentive if providers offer an intervention (eg. telephone call, referral to another healthcare provider or service, etc.).

Additionally for both groups, critical absolute scores or decrement in subsequent scores as measured by the aforementioned questionnaires will trigger an automated secured email alert to the patient’s treating MS provider. Critical scores and absolute change in scores considered to be meaningful were determined via analysis of normative data associated with individual PROMs (in a validated population of MS patients when available) (see Supplementary Appendix 1).

All groups will also be asked to complete the Consultation Satisfaction Questionnaire (CSQ) [29] as a measure of provider satisfaction, and the CollaboRATE survey [30] as a measure of shared decision-making, at baseline, and at end of the study at 12 months. At the end of the study, providers will be asked to complete a Provider Exit Survey in regards to their own perception on usage of the study measures in PwMS (Supplementary Fig. 4).

### Outcomes

The primary outcome of this study will be:

- The difference in change in HADS score between the intervention and conservative group at 12 months.

The secondary outcomes of this study will be:

- The difference in change in the CollaboRATE shared decision-making survey score and CSQ score between the intervention and the conservative groups at 12 months.
- Provider exit survey responses at 12 months.

### Data collection

The questionnaires will be delivered electronically to the patient at baseline for both conservative and intervention groups. For patients in the intervention group, questionnaires will be delivered electronically again at 6 months and at the end of the study at 12 months; in the conservative group, questionnaires will be delivered electronically again at the end of the study at 12 months. Initial CSQ and CollaboRATE surveys will be sent to both groups at baseline and at the end of the study. Patients will be sent an email to ask them to complete the questionnaires, with 2 follow-up reminders (1 week and 2 weeks) after the initial electronic contact. For provider exit surveys, mean and median values will be calculated from questions assessed on a Likert scale. Proportion of responses on each multiple choice answers will be determined on the one multiple choice question. Open ended responses will be assessed qualitatively.

Study methods, data management, and biostatical support is supplied by EPICORE Centre, Department of Medicine/Pharmacology, Faculty of Medicine and Dentistry (www.epicore.ualberta.ca). Data for the study will be collected and managed using REDCap at the University of Alberta. REDCap is a secure, web-based software platform designed to support data capture for research studies, providing 1) an intuitive interface for validated data capture; 2) audit trails for tracking data manipulation and export procedures; 3) automated export procedures for seamless data downloads to common statistical packages; and 4) procedures for data integration and interoperability with external sources [31, 32]. Data from administered PROMs through REDCap will allow for centralized data collection capable of export for future analysis.

### Sample size

Using the validation information in our previous study [23] and the following assumptions of 80% power and a two-sided alpha of 0.05, a total sample size of 356 (178 in each group) will be required to detect effect of 0.298 difference between the intervention and the conservative groups. The same size has been calculated for both HADS-Anxiety score (HADS-A) and HADS-Depression score (HADS-D) by the independent T-test. This study will use the sample size calculated for HADS-A, as it required a larger sample size and to ensure there will be sufficient power for both HADS-A and HADS-D. This sample size will be inflated by 10% to 392 (196 in each group) to account for possible dropouts, losses to follow-up, and withdrawals of consent.

### Data analysis

Prior to conducting statistical analysis, preliminary screening will be conducted using Statistical Analysis System (SAS) 9.4 software (SAS Institute Inc, Cary, NC, USA) to ensure that all the enrolled patients meet the eligibility inclusion and exclusion criteria and confirm the participants provide informed consent. Data assessors will be blinded to participant intervention versus conservative grouping status via de-identified data.

Data analysis will be performed using the computer R 3.4.0 software (Vienna, Austria; https://www.R-project.org/) and SAS 9.4 software. Patient demographic and clinical characteristics will be analysed using descriptive statistics. Categorical variables will be reported using frequency, and percentage and continuous variables will be reported using mean (standard deviation [SD]) or median (interquartile range [IQR]) as appropriate. Univariate level analysis will be conducted to determine if there is a statistical significance between the outcomes (e.g., baseline to six months and 12 months, respectively). Chi-square and Independent T-tests will be used for the univariate analysis, or where appropriate, non-parametric tests (Fishers test and Wilcoxon rank test) will be utilized. Multivariate analysis of variance (MANOVA) will be used to test for overall differences between the intervention and conservative groups at the different time points and variances amongst the variables by groups and time points. All test assumptions will be checked during the data analysis process. Statistical significance will be set at *p* values less than 0.05.

The primary outcome of difference in change of HADS-D and HADS-A, from baseline to 12 months between the intervention and conservative groups, will be tested using an independent T-test. The change of HADS score will be tested by MANOVA for two-way design setting to check if a statistically significant mean of change exists through time points between the groups. The MANOVA helps control for inflation of Type I error (rejection of a true null hypothesis as the result of a test procedure) and count the correlation between sections. Post-hoc Tukey Honestly Significant Difference (HSD) Test will be performed afterwards with adjustment by Bonferroni method, which allows many comparison statements to be made while still assuring an overall confidence coefficient is maintained. Paired T-test will also be considered for use. Regression analysis will be performed to quantify the change of CSQ through time points and between the groups considering patients’ individual effect as random effect. With more than 10% cumulative missing rate, the last observation will be carried forward in the case of missing data. The difference in change in both CSQ score and CollaboRATE shared decision-making survey score, will be analysed using the same methods as described above. The CSQ will be treated as continuous variables with an overall satisfaction score as a sum of the sub-scales for each question in the CSQ.

Linear mixed effect model will be used in multivariable analysis level in order to adjust patients’ individual effect and to quantify the change of outcome measure through time points and between the groups considering the impact of selected variables when they cooperate by presenting the coefficient (standard error), 95% confidence interval, and *P*-values. The variables included in the multivariable analysis will be selected if they were statistically significant on the univariate level analysis and considered clinically significant by the research team.

For provider exit surveys, mean and median values will be calculated from questions assessed on a Likert scale. Proportion of responses on each multiple choice answers will be determined on the one multiple choice question. Open ended responses will be assessed qualitatively.

## Discussion

Alberta has one of the highest incidences of MS in Canada [33]. MS carries a huge financial burden on both individual and society levels [34–36]. Neurologists working with PwMS usually require a great amount of support from healthcare professionals, and administrators. Yet, despite this, PwMS can feel that they do not receive enough education or support from their healthcare providers in order to meet their needs [33, 37–40]. Moreover, the education and needs of MS patients change during the course of their illness [41]. This study addresses many of the unmet needs identified by PwMS: coping strategies, increased communication strategies with their healthcare providers, more timely and effective intervention for symptoms of MS, complications and urgent relapse assessment and management, in addition to reviewing strategies used by people with MS to control and treat their disease, and in helping them optimize their functioning.

The chosen PROMs (HADS, EQ5D, MFIS, PDDS, and PHQ-9) were selected to encompass commonly reported and clinically relevant symptoms expressed by PwMS [42, 43]. More specifically, the difference in change in depression and anxiety scores were chosen as the primary outcome due to previous meta-analyses demonstrating depression and anxiety as responsive to intervention in PwMS [44–46].

Use of PROMs empowers PwMS to partner in their care with their MS healthcare providers. Patient engagement is a common goal between PwMS and providers to move towards shared decision-making, with electronic tools, patient-driven data, and access to high quality information identified as factors towards success [18, 19, 47]. Cross-sectional analyses in primary care have demonstrated increased patient activation as associated with reduced probability of ED visits, hospitalization, obesity, and smoking, along with increased likelihood of up to date status of age-appropriate cancer screening, normal blood pressure, and lipid laboratory markers [48]. Patient engagement is feasible, effective, and relevant in chronic medical conditions such as hypertension [49], diabetes [50], and heart failure [51]. In PwMS, higher patient activation is associated with improved depressive symptoms, quality of life, self-efficacy, and fatigue [46, 52, 53] as well as greater confidence in selecting disease modifying therapies in partnership with their MS healthcare providers [54]. A focus on patient engagement is a relatively novel topic with PwMS, but there was consensus among the Multiple Sclerosis in the 21^st^ Century Steering Committee (an international working group of both MS experts and patient group representatives) that integration of PROMs into clinical practice may be a means to facilitate patient engagement [22].

Quality of life in PwMS arguably is more representative of patients’ perspectives of their well-being in the context of their disease, and is influenced by comorbid depression, fatigue, and pain in addition to psychosocial determinants of health such as level of education and access to social supports [4, 55–57]. The mainstay of MS treatment is disease modifying therapies – medications typically targeting the immune system to slow or prevent ongoing central nervous system inflammation, both clinically and radiologically [58]. The impact of disease modifying therapy primarily manifests in prevention of relapses and accrual of neurological disability over time [59], but data is mixed when it comes to improving quality of life metrics, even with high efficacy second-line therapies [60]. Emphasis on covert symptoms such as depression and anxiety, pain, and fatigue has increased in recent years (as opposed to “hard” symptoms like hemiparesis and coordination), but only a modest amount of research lacking head-to-head trials exists exploring comprehensive intervention and treatment of these domains in PwMS [61–63]. Although difficult to separate confounders, evidence suggests interplay between these symptoms, and early treatment of one symptom may lead to improvement in others [64]. Consciously addressing day-to-day symptoms is crucial in comprehensive care and complements effective disease modifying therapies to optimize long term functioning; both are mutually interdependent and equally important to regularly discuss with patients to improve quality of life.

From the perspective of PwMS, their experience may not be fully captured from standardized surveys and questionnaires which comprise the majority of PROMs. Implementation and integration of the 3IP among interval measurement of PROMs allows participants in our study to communicate personalized, narrative information impactful in their ongoing care. Interviews with PwMS demonstrate the desire for effective communication with their MS provider at all stages of disease from diagnosis through to palliative care [65], and incorporation of the 3IP is a simple, relatively labour-free strategy of communication compared to a clinical interview or telephone call, and driven by each patient’s individualized needs. Thus, the addition of the 3IP will complement data collected from regular PROMs, and on a patient by patient basis, could be as or even more influential in ongoing personalized care of PwMS.

This study has potential limitations. Firstly, both patients and providers are required to participate on a longitudinal basis for a minimum of 12 months. For patients, automatically generated email reminders will prompt them to complete their repeat PROMs, but ultimately they must decide to participate on a continuing basis. On a related note, the decision to set the difference in frequency of PROM collection at 6 months and 12 months for interventional and conservative groups, respectively, was based on pragmatic implementation of the study in a clinical setting. The frequency of PROM administration for the conservative group at 12 months reflects standard annualized clinical follow up for stable PwMS at our centre, in line with international [66] and Canadian guidelines [67]. Setting PROM collection frequency at 6 months for the intensive group was preferable from a practical perspective as frequent enough to capture critical PROM scores and changes between annual visits, but not too frequent as to result in disengagement from PwMS or providers. Additionally, while designed to be accessible to incorporate into workflow, monitoring patient PROMs can only be performed by accessing REDCap, which is separate from EMRs in clinical use at our sites. This will add time and cognitive labour for providers, and an increased frequency of PROM collection may present a practical barrier to clinical use [68]. By setting PROM collection for the interventional group at 6 months, this will ideally allow a more protracted timeframe to integrate a novel clinical tool for providers.

Providers will additionally need to determine how to incorporate data gathered from PROMs, critical alerts, and the 3IP into their daily workflow and patient management; consequently, uptake and utilization of PROM information into day-to-day practice by individual providers may be heterogenous. The use of PROMs may work better when integrated directly into the patients’ electronic medical records system (EMR). Different Alberta clinics use different electronic medical records systems, and the functionality of integrating such PROMs prompts into each EMR system was beyond the scope of this study, as we wished to examine the utility of regular PROM use in different outpatient MS health provider settings (ie: tertiary MS clinic, community MS clinic, private practice neurologists’ offices). Therefore, there may be an extra step in opening up the EMR chart for the patients for the healthcare providers when receiving a secure email alert about completion of PROMs. Furthermore, survey administration, data collection, and alert notification via REDCap on a digital-only format aids in execution of our study, but potentially may limit accessibility and enduring follow-up for less technologically-savvy participants.

In conclusion, this randomized controlled trial will explore the feasibility of incorporating systematic PROM completion by patients and hopefully, will build on the existing communication channels between MS healthcare providers and PwMS. Finally, this study will evaluate whether the systematic collection of PROMs enhances or alters depression or anxiety levels in PwMS. This study will also secondarily examine the influence of regular implementation of PROMs on the perception of provider satisfaction and shared decision making by PwMS, and resultant qualitative provider responses and interventions on the utility and feasibility of PROMs in their MS practice. The results of this study may add to the growing literature focused on PROMs in PwMS, and delineate the practicality of their use with both PwMS and their healthcare providers.

## Trial status

This trial was registered on July 28, 2021 at the National Institutes of Health United States National Library of Medicine, ClinicalTrials.gov NCT04979546. Recruitment started in November 4, 2021 and the first participant was enrolled on the same day. At the time of manuscript submission, a total of 191 unique participants were enrolled into the study. The estimated completion date will be December 2023.

## Supplementary Information

**Additional file 1: Supplementary Figure 1.** Advertisement flyer for patient recruitment. Patients are linked to further information study, informed consent, and self-registration pages on REDCap via URL code (https://redcap.link/PROMsInMS) or QR code. MS, multiple sclerosis.

**Additional file 2: Supplementary Figure 2.** Example informed consent form for participants. Participants access the form directly via REDCap. MS, multiple sclerosis; PROMs, patient reported outcome measures.

**Additional file 3: Supplementary Figure 3.** Example of Provider dashboard of a patient in the Intervention group. Providers can view PROM and 3IP information on their patients, depending on their randomization, and document their responses after viewing. Providers can also see PROM values in relation to predetermined critical values. PROM, patient reported outcome measure; 3IP, 3-item prompt; PHQ-9, Patient Health Questionnaire-9; PDDS, Patient Determined Disease Steps; MFIS, Modified Fatigue Impact Scale; HADS, Hospital Anxiety and Depression Scale; EQ-5D, EuroQol five-dimensional questionnaire.

**Additional file 4: Supplementary Figure 4.** Provider exit survey. KEC, Kaye Edmonton Clinic; PROMs, patient reported outcome measures; PwMS, persons with multiple sclerosis.

**Additional file 5: Supplementary Appendix 1.** PROMs descriptions, and critical scores and absolute change in scores triggering alert to providers. EQ5D, EuroQoL-5D; VAS, Visual Analogue Scale; PwMS, persons with multiple sclerosis; SD, standard deviation; IQR, interquartile range; MFIS, Modified Fatigue Impact Scale; MS, multiple sclerosis; HADS, Hospital Anxiety and Depression Scale; PDDS, Patient Determined Disease Steps; FS, Functional System; EDSS, Expanded Disability Status Scale; PHQ-9, Patient Health Questionnaire.

**Additional file 6: Supplementary Appendix 2.** World Health Organization Trial Registration Data Set.

## Data Availability

Not applicable – data will not be shared publicly due to sensitive personal information and patient confidentiality. Once the trial is completed, publication of results through an academic journal will be pursued.

## Abbreviations

3IP: 3-Item prompt
CSQ: Consultation Satisfaction Questionnaire
DAG: Data Access Group
EQ5D: EuroQol five-dimensional questionnaire
HADS-A: Hospital Anxiety and Depression Scale – Anxiety Score
HADS-D: Hospital Anxiety and Depression Scale – Depression Score
HADS: Hospital Anxiety and Depression Scale
HSD: Honestly Significant Difference
IQR: Interquartile range
MANOVA: Multivariate analysis of variance
MFIS: Modified Fatigue Impact Scale
MS: Multiple sclerosis
PDDS: Patient Determined Disease Steps
PHQ-9: Patient Health Questionnaire-9
PROMs: Patient-reported outcome measures
PwMS: Persons with multiple sclerosis
QoL: Quality of life
QR: Quick Response
REDCap: Research Electronic Data Capture
SAS: Statistical Analysis System
SD: Standard deviation
SPIRIT: Standard Protocol Items: Recommendations for Interventional Trials
URL: Uniform Resource Locator
