# Supplementary material for: Evaluating the impact of patient-reported outcome measures on depression and anxiety levels in people with multiple sclerosis: a study protocol for a randomized controlled trial
Source: BMC Neurol. 2023 Feb 2;23:53. doi: 10.1186/s12883-023-03090-0 (PMC9893570; doi:10.1186/s12883-023-03090-0)
Supplement: Supplementary file 1 — Additional file 1: Supplementary Figure 1. Advertisement flyer for patient recruitment. Patients are linked to further information study, informed consent, and self-registration pages on REDCap via URL code (https://redcap.link/PROMsInMS) or QR code. MS, multiple sclerosis. [file 12883_2023_3090_MOESM1_ESM.pdf]

University of Alberta Ethics # Pro00111593

LIVING WITH MS?

You may be eligible for a study being done by Dr. Penny Smyth at the University of Alberta involving persons with multiple sclerosis.

You May Qualify If You:

- Are over 18 years old
- Have been diagnosed with multiple sclerosis by a neurologist
- Have regular appointments with a neurologist in Edmonton or Red Deer for your multiple sclerosis
- Have access to a computer or smartphone with internet access

Participation involves:

- Completing an online survey (about 30-45 minutes), repeated once or twice over 12 months
- Attending regular appointments with your neurologist as usual

FOR MORE INFORMATION

Visit **https://redcap.link/PROMsInMS** or **scan in the QR code with your smartphone camera** to read more about the study and for registration.

If you have further questions, you can contact Dr. Nathan Chu at nathan.chu@albertahealthservices.ca

University Hospital Foundation
