# Supplementary material for: Evaluating the impact of patient-reported outcome measures on depression and anxiety levels in people with multiple sclerosis: a study protocol for a randomized controlled trial
Source: BMC Neurol. 2023 Feb 2;23:53. doi: 10.1186/s12883-023-03090-0 (PMC9893570; doi:10.1186/s12883-023-03090-0)
Supplement: Supplementary file 2 — Additional file 2: Supplementary Figure 2. Example informed consent form for participants. Participants access the form directly via REDCap. MS, multiple sclerosis; PROMs, patient reported outcome measures. [file 12883_2023_3090_MOESM2_ESM.pdf]

PARTICIPANT INFORMATION SHEET

Title of Study: Using Patient-Reported Outcomes To Improve the Care of People with Multiple Sclerosis: A randomized trial.

Principal Investigator: Penny Smyth, MD, FRCPC – Associate Professor, Division of Neurology, Department of Medicine, Faculty of Medicine & Dentistry, University of Alberta

Co-investigator: Ross T. Tsuyuki, BSc(Pharm), PharmD, MSc, FCSHP, FACC, FCAHS, ISHF - Professor and Chair, Department of Pharmacology, Professor of Medicine (Cardiology) and Director, EPICORE Centre, Faculty of Medicine & Dentistry, University of Alberta

Research/Study Coordinator: Nathan Chu, MD – Senior Neurology Resident, Division of Neurology, Department of Medicine, Faculty of Medicine & Dentistry, University of Alberta

---

Why am I being asked to take part in this research study?

You are being asked to participate in this study as a person with multiple sclerosis (MS) being treated by a neurologist in northern Alberta. The purpose of this study is to investigate the effect of using patient-centered questionnaires in persons with MS. The current standard of care does not involve regular use of these tools, and we hope the results of this study will help us care for persons with MS better.

This sheet gives you information about what happens if you choose to participate, and how your information will be handled and protected. Before you make a decision, read this information sheet carefully at your own pace. You will have the option of printing a copy of this form for your records.

What is the reason for doing the study?

MS is a complex disease affecting people in many different ways. When a doctor talks to a person with MS about their symptoms, examines them, and orders brain scans and tests, that is only a small part of what someone experiences. It is becoming clearer that a person's own thoughts of how their disease affects their life plays an important role in their health. Surveys and questionnaires that measure how a person experiences their disease are called Patient Related Outcome Measures (PROMs).

We do not regularly use these PROMs at this time in persons with MS. Therefore, we do not know if using them has an impact on care in persons with MS.

What will I be asked to do?

If you agree to participate, you will be emailed a secure link to complete surveys on how MS affects your life. You will also fill out a brief response to the question "What are the top 3 things you would like your MS Neurologist to know about you right now?" The entirety of the surveys should take about 30-45 minutes. They can be completed on a computer or smartphone with internet access. You will be

asked to repeat these surveys either 1 or 2 times in a 12 month period, depending on which research group you are assigned.

You will be assigned at random to 1 of 2 groups. You will have a 50% chance of being in the intensive treatment group and 50% chance of being in the standard treatment group.

In the intensive treatment group, you will be asked to fill out the surveys at three time points: at start of the study, at 6 months, and at the end of the study at 12 months. Your neurologist will have access to the scores from your surveys and response to your “Top 3 things for your neurologist to know” in this group.

In the standard treatment group, you will be asked to fill out the PROM surveys at two time points: at start of the study, and at the end of the study at 12 months Your neurologist will **not** have access to the scores from your surveys in this group. However, he/she will be able to see your response to your “Top 3 things for your neurologist to know”.

For both groups, if your survey scores are below a pre-set score, your neurologist will be notified by a secured message. You may then be contacted by him/her office.

What are the risks and discomforts?

Completing the PROM surveys will need 30-45 minutes of concentration. This may result in mental or psychological fatigue. If this occurs, you are free to return to the surveys at after a short break.

In completing the PROM surveys, you may uncover symptoms related to depression, anxiety, fatigue, or decreased quality of life. Again, you are free to return to the surveys after a short break. If your survey scores are below a pre-set score, your neurologist will be notified by a secured message. You may then be contacted by his/her office.

Overall, we do not think there are greater risks or discomforts in participating in this study than standard of care.

It is not possible to know all of the risks that may happen in a study, but the researchers have taken all reasonable safeguards to minimize any known risks to a study participant. If we find out anything new during the course of this research which may change your willingness to be in the study, we will tell you about these findings.

What are the benefits to me?

Participating in this study may provide increased communication strategies with your healthcare providers. It may also provide more timely intervention for your symptoms of MS or complications. Also, completing PROM surveys may allow participants to gain greater awareness into their own disease. This study may help us provide better care for other persons with MS in the future. However, you may not get any benefit from being in this research study.

Do I have to take part in the study?

Being in this study is your choice. If you decide to be in the study, you can change your mind and leave the study at any time. This will in no way affect the care, treatment, or services that you are entitled to.

You may also choose to only participate in parts of the survey and are not required to answer any questions for any reason.

You may request to withdraw from the study at any time. You can contact us at any of the phone numbers and email addresses listed above, and request to opt out of the study. Your data and information will be excluded from the study. This can be requested up until we finish collecting data for this study (projected to be around September 2022).

Will my information be kept private?

During the study we will be collecting data about you from the surveys you complete. We will do everything we can to make sure that this data is kept private. Your data will be kept on secured and encrypted online servers and computers. No data relating to this study that includes your name will be released outside of the researcher's office or published by the researchers. Sometimes, by law, we may have to release your information with your name so we cannot guarantee absolute privacy. However, we will make every legal effort to make sure that your information is kept private.

The researchers will **not** be accessing your medical records as part of this study, and the information we collect from you will be kept confidential in a secured, encrypted server.

Depending on which group you are in, some or all of your PROM survey information will be available via a secured, encrypted online database to your neurologist. For both groups, if your scores on your survey are at a certain level, your neurologist will be notified by a secured message, and you may be contacted by his/her office.

After the study is done, we will securely store the data that was collected as part of the study. At the University of Alberta, we keep data stored for a minimum of 5 years after the end of the study. If you leave the study, we will not collect new health information about you, but we may need to keep the data that we have already collected.

With your permission, we will store your contact information (name, email address, phone number) for possible contact in the future for other studies involving persons with MS. This is not mandatory, and you can opt out in the consent form below.

During research studies it is important that the data we get is accurate. For this reason your health data, including your name, may be looked at by auditors from the University Hospital Foundation/University of Alberta, and the Health Research Ethics Board.

What if I have questions?

If you have any questions about the research now or later, please contact our research team at the email address [redacted]

If you have any questions regarding your rights as a research participant, you may contact the Health Research Ethics Board at 780-492-2615. This office has no affiliation with the study investigators.

This study was funded through the University Hospital Foundation as part of ongoing research efforts to look at strategies to improve care for people with MS. This clinical trial has been registered at *clinicaltrials.gov* under the registration number NCT04979546.

Should you wish to participate in our study, please fill out the enclosed consent form.

PARTICIPANT CONSENT FORM

Title of Study: Using Patient-Reported Outcomes in the Care of People with Multiple Sclerosis: A randomized trial.

Principal Investigator: Penny Smyth, MD, FRCPC – Associate Professor, Division of Neurology, Department of Medicine, Faculty of Medicine & Dentistry, University of Alberta

Co-investigator: Ross T. Tsuyuki, BSc(Pharm), PharmD, MSc, FCSHP, FACC, FCAHS, ISHF - Professor and Chair, Department of Pharmacology, Professor of Medicine (Cardiology) and Director, EPICORE Centre, Faculty of Medicine & Dentistry, University of Alberta

Research/Study Coordinator: Nathan Chu, MD – Senior Neurology Resident, Division of Neurology, Department of Medicine, Faculty of Medicine & Dentistry, University of Alberta

	Yes	No
Do you understand that you have been asked to be in a research study?	☐	☐
Have you read and received a copy of the attached Information Sheet?	☐	☐
Do you understand the benefits and risks involved in taking part in this research study?	☐	☐
Do you understand at any time you can email the research coordinator at nathan.chu@albertahealthservices.ca to ask questions and discuss the study further?	☐	☐
Do you understand that you are free to leave the study at any time, without having to give a reason and without affecting your future medical care?	☐	☐
Do you understand that your study records/information will only be accessed by the research team, or auditors from the University Hospital Foundation/University of Alberta, and the Health Research Ethics Board, but otherwise be kept confidential?	☐	☐
Do you agree to be contacted for follow-up or to facilitate future research?	☐	☐

I agree to take part in this study:

____________________________ ____________________________

Signature of participant Printed name

Date: ____________________________
