# Supplementary material for: Evaluating the impact of patient-reported outcome measures on depression and anxiety levels in people with multiple sclerosis: a study protocol for a randomized controlled trial
Source: BMC Neurol. 2023 Feb 2;23:53. doi: 10.1186/s12883-023-03090-0 (PMC9893570; doi:10.1186/s12883-023-03090-0)
Supplement: Supplementary file 3 — Additional file 3: Supplementary Figure 3. Example of Provider dashboard of a patient in the Intervention group. Providers can view PROM and 3IP information on their patients, depending on their randomization, and document their responses after viewing. Providers can also see PROM values in relation to predetermined critical values. PROM, patient reported outcome measure; 3IP, 3-item prompt; PHQ-9, Patient Health Questionnaire-9; PDDS, Patient Determined Disease Steps; MFIS, Modified Fatigue Impact Scale; HADS, Hospital Anxiety and Depression Scale; EQ-5D, EuroQol five-dimensional questionnaire. [file 12883_2023_3090_MOESM3_ESM.pdf]

Provider Exit Survey
**** All responses will be anonymized.***

1. For the duration of this study, my MS practice was primarily located in:
   ☐ Northern Alberta MS Clinic (KEC) ☐ Edmonton and Red Deer Community Clinics

   ☐ Red Deer MS Clinic

2. For the following questions on using patient related outcome measures (PROMs) in persons with MS (PwMS), please select the response which best characterizes how you feel about the statement:

	Strongly Disagree	Disagree	Neutral	Agree	Strongly Agree
A. PROMs in PwMS were useful for patient care	1 ☐	2 ☐	3 ☐	4 ☐	5 ☐
B. PROMs in PwMS were harmful to the therapeutic relationship	1 ☐	2 ☐	3 ☐	4 ☐	5 ☐
C. I routinely accessed PROMs in PwMS during the study when prompted	1 ☐	2 ☐	3 ☐	4 ☐	5 ☐
D. I routinely took action/checked in on patients after accessing PROMs in PwMS during the study	1 ☐	2 ☐	3 ☐	4 ☐	5 ☐
E. I routinely took action/ checked in on patients after being alerted to **critical values** of PROMs in PwMS during the study	1 ☐	2 ☐	3 ☐	4 ☐	5 ☐
F. I thought routine PROM in PwMS improved patient care	1 ☐	2 ☐	3 ☐	4 ☐	5 ☐
G. PROMs in PwMS were easy to incorporate into patient care	1 ☐	2 ☐	3 ☐	4 ☐	5 ☐

3. During this study, I found:

☐ PROM questionnaires were more useful than the open ended text answers
☐ Open ended text answers were more useful than PROM questionnaires
☐ Both PROM questionnaires and the open ended text answers were useful
☐ Neither PROM questionnaires and the open ended text answers were useful

4. Any other comments?:
