# Supplementary material for: Evaluating the impact of patient-reported outcome measures on depression and anxiety levels in people with multiple sclerosis: a study protocol for a randomized controlled trial
Source: BMC Neurol. 2023 Feb 2;23:53. doi: 10.1186/s12883-023-03090-0 (PMC9893570; doi:10.1186/s12883-023-03090-0)
Supplement: Supplementary file 4 — Additional file 4: Supplementary Figure 4. Provider exit survey. KEC, Kaye Edmonton Clinic; PROMs, patient reported outcome measures; PwMS, persons with multiple sclerosis. [file 12883_2023_3090_MOESM4_ESM.pdf]

Randomization Group: Intervention

3 things to know about patient:

1: I work very hard to stay healthy and strong

2: I am able to work full time and manage symptoms by working out and doing some natural therapies

3: I have very supportive family and friends

Patient's scores:

PHQ-9 Score:
4
(Score of >= 10 is critical)

PDDS Score:
0
(Score of >= 3 is gait impairment)

MFIS Score:
42
(Score of >= 58 is critical)

HADS Score:
7
(Score of >= 11 is critical)

EQ-5D Score:
0.89
(Score of <= 0.48 is critical)

Provider Response:

Provider response, if applicable

- [ ] Phoned patient
- [ ] Scheduled earlier appointment.
- [ ] Changed/altered medications.
- [ ] Made a referral to another provider (allied health or another specialty), specify
- [x] Other, specify

Specify other
* must provide value

messaged patient to reassure her that her scores are good.
