# Supplementary material for: Evaluating the impact of patient-reported outcome measures on depression and anxiety levels in people with multiple sclerosis: a study protocol for a randomized controlled trial
Source: BMC Neurol. 2023 Feb 2;23:53. doi: 10.1186/s12883-023-03090-0 (PMC9893570; doi:10.1186/s12883-023-03090-0)
Supplement: Supplementary file 5 — Additional file 5: Supplementary Appendix 1. PROMs descriptions, and critical scores and absolute change in scores triggering alert to providers. EQ5D, EuroQoL-5D; VAS, Visual Analogue Scale; PwMS, persons with multiple sclerosis; SD, standard deviation; IQR, interquartile range; MFIS, Modified Fatigue Impact Scale; MS, multiple sclerosis; HADS, Hospital Anxiety and Depression Scale; PDDS, Patient Determined Disease Steps; FS, Functional System; EDSS, Expanded Disability Status Scale; PHQ-9, Patient Health Questionnaire. [file 12883_2023_3090_MOESM5_ESM.pdf]

Appendix 1: Patient related outcome measures (PROMs) and triggering alert thresholds

1. EQ5D – EuroQoL-5D (1)
    a. Quality of life measure, using 5 dimensions of health (Mobility, Self-Care, Usual Activities, Pain/Discomfort, Anxiety/Depression)
    b. Score converted to index value based on different regional values
        i. 3L and 5L versions in which there are either 3 or 5 levels of responses to each category
    c. Employs a Visual Analogue Scale (VAS) which patients rate their health from 0-100 (100 being the best possible score)
    d. Validated with specific data set measured in PwMS (2)
    e. Triggers for alerting
        i. EQ5D index value ≤ 0.48 at any time – ie. Mean – 1 SD to capture those below the 16th percentile
        ii. Decrease in index value ≥ 0.26 on subsequent testing – ie. 1 SD decrease – to capture those with a substantial decrease in their score over time.
        iii. VAS value ≤ 60 at any time (ie. 25th percentile or lower, as determined by the IQR 60.00-88.00 in the above data) – to capture those patients with subjectively decreased QoL perhaps not elicited by specific domains
2. MFIS - Modified Fatigue Impact Scale (3)
    a. 21-item questionnaire with 3 subsections – Physical, Cognitive and Psychosocial
        i. Subscales have individual ranges of scoring
            - Physical – 0 to 36
            - Cognitive – 0 to 40
            - Psychosocial – 0 to 8
        ii. Total score of 0 to 84
    b. MS patient specific normative data previously measured (4)
    c. Triggers for alerting
        i. Absolute total score ≥ 58 at any time – ie. Mean + 1 SD – to capture those above the 84th percentile in score.
        ii. Increase in absolute total score ≥ 17 on subsequent testing – ie 1 SD – to capture those with a substantial increase in their score over time.

3. HADS – Hospital Anxiety and Depression Scale (5)
    a. 14-item questionnaire divided into 7 questions each related to either depression or anxiety symptoms
        i. Scores range from 0 to 21 for either depression or anxiety, with <u>11 to 21 in one category considered "Moderate" or "Severe"</u>
    b. MS patient specific data from Jones et al, 2012 (6)
    c. Triggers for alerting
        i. Absolute score ≥ 11 in either depression or anxiety category at any time
        ii. Increase in depression score ≥ 4 on subsequent testing – ie. 1 SD increase
        iii. Increase in anxiety score ≥ 4 on subsequent testing – ie. 1 SD increase
4. PDDS - Patient Determined Disease Steps (7)
    a. Measures handicap in eight neurological domains: mobility, hand function, vision, fatigue, cognition, bladder/bowel, sensory and spasticity
    b. Domains are almost entirely equivalent with the FS of the EDSS, with the main difference that the cerebellar function is not directly represented on the PDDS, even though it is reflected on the mobility and hand function
    c. Triggers for alerting
        i. PDDS ≥ 3 at first testing – trigger to notify with gait impairment but not requiring aid yet;
            - PDDS 3 is about the equivalent of EDSS of 4.5 when gait is beginning to be impaired but aids not needed (8)
        ii. Increase of score ≥ 1 on subsequent measurements – any increase is functionally meaningful and clinically significant
5. PHQ-9 – Patient Health Questionnaire (9)
    a. Depression screening tool
    b. Validated in neurological and MS patients as sensitive and specific
        i. Validation in neurological illness – <u>cut off point of 10 valid as positive screen</u> (10)
        ii. Measured in MS populations specifically – <u>cut off point of 10 valid</u> (although study argued that 11 is reasonable, no strong evidence to displace the standard cut off of 10) (11)

c. Triggers for alerting
    i. Score of ≥ 10 at any time
    ii. Increase in score by ≥ 6 on subsequent testing – ie 1 SD increase to capture patients deteriorating, but not necessarily reaching absolute score of ≥ 10

Appendix References

1. EuroQol G. EuroQol--a new facility for the measurement of health-related quality of life. Health Policy. 1990;16(3):199-208.
2. McKay KA, Ernstsson O, Manouchehrinia A, Olsson T, Hillert J. Determinants of quality of life in pediatric- and adult-onset multiple sclerosis. Neurology. 2020;94(9):e932-e41.
3. Fisk JD, Pontefract A, Ritvo PG, Archibald CJ, Murray TJ. The impact of fatigue on patients with multiple sclerosis. Can J Neurol Sci. 1994;21(1):9-14.
4. Strober LB, Bruce JM, Arnett PA, Alschuler KN, DeLuca J, Chiaravalloti N, et al. Tired of not knowing what that fatigue score means? Normative data of the Modified Fatigue Impact Scale (MFIS). Mult Scler Relat Disord. 2020;46:102576.
5. Zigmond AS, Snaith RP. The hospital anxiety and depression scale. Acta Psychiatr Scand. 1983;67(6):361-70.
6. Jones KH, Ford DV, Jones PA, John A, Middleton RM, Lockhart-Jones H, et al. A large-scale study of anxiety and depression in people with Multiple Sclerosis: a survey via the web portal of the UK MS Register. PLoS One. 2012;7(7):e41910.
7. Rizzo MA, Hadjimichael OC, Preiningerova J, Vollmer TL. Prevalence and treatment of spasticity reported by multiple sclerosis patients. Mult Scler. 2004;10(5):589-95.
8. Learmonth YC, Motl RW, Sandroff BM, Pula JH, Cadavid D. Validation of patient determined disease steps (PDDS) scale scores in persons with multiple sclerosis. BMC Neurol. 2013;13:37.
9. Kroenke K, Spitzer RL, Williams JB. The PHQ-9: validity of a brief depression severity measure. J Gen Intern Med. 2001;16(9):606-13.
10. Williams KG, Sanderson M, Jette N, Patten SB. Validity of the Patient Health Questionnaire-9 in neurologic populations. Neurol Clin Pract. 2020;10(3):190-8.
11. Patten SB, Burton JM, Fiest KM, Wiebe S, Bulloch AG, Koch M, et al. Validity of four screening scales for major depression in MS. Mult Scler. 2015;21(8):1064-71.
