# Supplementary material for: Evaluating the impact of patient-reported outcome measures on depression and anxiety levels in people with multiple sclerosis: a study protocol for a randomized controlled trial
Source: BMC Neurol. 2023 Feb 2;23:53. doi: 10.1186/s12883-023-03090-0 (PMC9893570; doi:10.1186/s12883-023-03090-0)
Supplement: Supplementary file 6 — Additional file 6: Supplementary Appendix 2. World Health Organization Trial Registration Data Set. [file 12883_2023_3090_MOESM6_ESM.pdf]

WHO Trial Registration Data Set (Version 1.3.1)

1. **Primary Registry and Trial Identifying Number**
   The National Institutes of Health United States National Library of Medicine, ClinicalTrials.gov. NCT04979546 – accessible from https://clinicaltrials.gov/ct2/show/NCT04979546
2. **Date of Registration in Primary Registry**
   July 28, 2021.
3. **Secondary Identifying Numbers**

   University of Alberta Research Ethics Board Approval – Project Pro00111593
   University Hospital Foundation (University of Alberta) – Award #18499
   Sanofi Canada – Award #0000201382
4. **Source(s) of Monetary or Material Support**
   University Hospital Foundation (University of Alberta) – Award #18499
   Sanofi Canada – Award #0000201382
5. **Primary Sponsor**
   University Hospital Foundation (University of Alberta) – Award #18499
6. **Secondary Sponsor(s)**
   Sanofi Canada – Award #0000201382

7. **Contact for Public Queries**
   Principal Investigator: Penelope Smyth, MD, FRCPC – Associate Professor, Division of Neurology, Department of Medicine, Faculty of Medicine & Dentistry, University of Alberta

   Clinical Sciences Building, 8440 112 Street NW, Edmonton, AB Canada T6G 2B7

   Ph:780-248-1775

   Email: smyth@ualberta.ca
8. **Contact for Scientific Queries**
   Principal Investigator: Penelope Smyth, MD, FRCPC – Associate Professor, Division of Neurology, Department of Medicine, Faculty of Medicine & Dentistry, University of Alberta

   Clinical Sciences Building, 8440 112 Street NW, Edmonton, AB Canada T6G 2B7

   Ph:780-248-1775

   Email: smyth@ualberta.ca

9. **Public Title**
   Using Patient-Reported Outcomes To Improve the Care of People With Multiple Sclerosis

10. **Scientific Title**
    Evaluating the impact of patient-reported outcome measures on depression and anxiety levels in people with multiple sclerosis: a randomized controlled trial.
11. **Countries of Recruitment**
    Canada
12. **Health Condition(s) or Problem(s) Studied**
    Multiple sclerosis, depression, anxiety
13. **Intervention(s)**
    Intervention group: All participants randomized to the intervention group will be asked to complete patient reported outcome measure (PROM) questionnaires at baseline, 6 months and 12 months (12 month study duration). Their treating neurologist will view the participant scores in addition to the text response to the 3-item prompt.

    Conservative group: those in the conservative group will complete the same questionnaires at baseline and at 12 months. Additionally, the treating neurologist will only be prompted to view the text response to the 3-item prompt, and will not be able to access the PROM questionnaire scores for participants in the conservative group.
14. **Key Inclusion and Exclusion Criteria**
    Inclusion criteria are:
    - A confirmed diagnosis of MS by a qualified healthcare provider. All subtypes falling under the diagnosis of multiple sclerosis including relapsing-remitting, secondary progressive, and primary progressive, among others, are eligible to participate.
    - Active patient of an Alberta-based neurologist/MS nurse practitioner.
    - Able/willing to complete informed consent and electronic PROM questionnaires.
    - Able to use a computer/smartphone.
    - English-speaking.

    Exclusion criteria are:
    - A suspected but not confirmed diagnosis of MS, a diagnosis of clinically/radiologically isolated syndrome, or a central nervous system inflammatory disorder other than MS.
    - PwMS not being managed by a participating neurologist/MS nurse practitioner.
    - Unwilling/unable to provide consent.
    - Unwilling/unable complete the electronic PROM questionnaires.
    - Cannot speak English.
    - Under the age of 18.
15. **Study Type**
    - Type of study: Interventional
    - Study design:

        - Method of allocation: Randomized
        - Masking: Single (Outcomes Assessor)
        - Assignment: Parallel
        - Allocation concealment and sequence generation: computer generated via Research Electronic Data Capture (REDCap) software at time of randomization.

16. **Date of First Enrollment**
    Enrolment of the first participant: November 4, 2021.

17. **Sample Size**
    - Number of participants that the trial plans to enrol in total: 396
    - Number of participants that the trial has enrolled: 191

18. **Recruitment Status**

    Recruiting: participants are currently being recruited and enrolled.

19. **Primary Outcome(s)**
    The primary outcome of this study will be the difference in change in Hospital Anxiety and Depression Scale (HADS) score between the intervention and conservative group at 12 months.

20. **Key Secondary Outcomes**
    The secondary outcomes of this study will be:
    - The difference in change in the CollaboRATE shared decision-making survey score and Consultant Satisfaction Questionnaire score between the intervention and the conservative groups at 12 months.
    - The proportion and type of healthcare provider intervention/alerts initiated by EuroQol five-dimensional questionnaire, Modified Fatigue Impact Scale, HADS, Patient Determined Disease Steps, or Patient Health Questionnaire-9 score at 12 months.
    - Provider exit survey responses at 12 months.

21. **Ethics Review**
    - Approved
    - Date of approval: August 6, 2021
    - University of Alberta Research Ethics Office (Health Research Ethics Board)
        - 2-01 North Power Plant (NPP, 11312 - 89 Avenue NW, Edmonton, AB T6G 2N2
        - Email: reoffice@ualberta.ca

22. **Completion date**
    To be determined; anticipated completion date of December 2023.

23. **Summary Results**
    Not applicable – study still in enrollment stage.

24. **IPD sharing statement**
    No current plans to share IPD data.
